# BRG1 exacerbates myocardial fibrosis after myocardial infarction by interacting with ZEB1

**DOI:** 10.3389/fphar.2026.1802700

**Published:** 2026-03-16

**Authors:** Yunfeng Cui, Jing Jin, Yingtao Cui, Ziyue Ma, Tingting Tong, Lisi Xiong, Meimei Shen, Yu Zhao, Xin Guo, Wen Liang, Hongxia Zhao, Tao Ban, Rong Huo

**Affiliations:** 1 Department of Pharmacology (State Key Laboratory of Frigid Zone Cardiovascular Diseases, Ministry of Science and Technology, State Key Laboratory-Province Key Laboratories of Biomedicine-Pharmaceutics of China, Key Laboratory of Cardiovascular Research, Ministry of Education), College of Pharmacy, Harbin Medical University, Harbin, China; 2 Department of Pharmacy, Harbin Medical University Cancer Hospital, Harbin, China; 3 Heilongjiang Academy of Medical Sciences, Harbin, China; 4 Department of General Surgery, The Fourth Affiliated Hospital of Harbin Medical University, Harbin, China

**Keywords:** BRG1, cardiac fibrosis, myocardial infarction, PP2A, ZEB1

## Abstract

**Background:**

Myocardial fibrosis, characterized by excessive collagen deposition and fibroblast activation, is a pivotal pathological process driving heart failure after myocardial infarction (MI). Our prior research revealed that Brahma-related gene 1 (BRG1) expression is elevated after MI and exacerbated cardiac electrophysiological remodeling; however, its precise role and molecular mechanism in post-MI fibrosis remain undefined.

**Methods:**

BRG1 expression was assessed in a mouse MI model and in TGF-β1-stimulated cardiac fibroblasts (CFs). Gain- and loss-of-function studies were performed using adenoviral vectors, siRNA, and plasmids *in vitro* and *in vivo*. Cardiac function and fibrosis were evaluated by echocardiography and histology. The molecular mechanism was dissected through co-immunoprecipitation (Co-IP), dual-luciferase reporter assays, chromatin immunoprecipitation (ChIP), and functional rescue experiments targeting the PP2A/Smad3 axis.

**Results:**

BRG1 was upregulated in fibrotic mouse hearts post-MI and in activated CFs. *In vivo*, BRG1 knockdown via AAV9-shRNA improved cardiac function, reduced infarct size, and attenuated fibrosis. *In vitro*, BRG1 promoted CFs proliferation, migration, and collagen production. Mechanistically, TGF-β1 enhanced the interaction between BRG1 and the transcription factor ZEB1. This complex transcriptionally repressed *Ppp2r1a*, the gene encoding the PP2A structural subunit Aα, leading to diminished PP2A activity. Consequently, Smad3 phosphorylation and nuclear translocation were enhanced, amplifying the pro-fibrotic TGF-β/Smad3 cascade. Crucially, ZEB1 knockdown or PP2A inhibition (okadaic acid) could respectively block or rescue the fibrotic effects of BRG1. Finally, BRG1 knockdown similarly suppressed fibrotic activation in human CFs.

**Conclusion:**

Our study defines a novel BRG1/ZEB1/PP2A transcriptional axis as a key driver of myocardial fibrosis and suggests BRG1 as a potential therapeutic target for mitigating fibrotic remodeling after MI.

## 1 Introduction

Cardiac fibrosis, a common pathological hallmark of diverse cardiovascular diseases such as diabetic cardiomyopathy, myocardial infarction (MI), and atherosclerosis (Armstrong et al., 1998), is primarily characterized by excessive collagen deposition, extracellular matrix (ECM) accumulation, and fibroblast hyperactivation and proliferation. These pathological changes drive cardiac dysfunction and maladaptive remodeling, ultimately culminating in heart failure (Travers et al., 2016; Gyongyosi et al., 2017). Cardiac fibroblasts (CFs) in connective tissue transform into myofibroblasts, resulting in the overproduction of α-smooth muscle actin (α-SMA), type I collagen (Col-I), and fibronectin (FN1), which are pivotal factors driving the progression of pathological fibrosis (Prabhu and Frangogiannis, 2016; Tallquist, 2020). It is vital to unravel the molecular mechanisms behind cardiac fibrosis to create targeted treatments.

Recombinant chromatin-associated Brahma-related gene 1 (BRG1) serves as the active enzyme component with ATPase function within the Switch/Sucrose Non-Fermentable Chromatin (SWI/SNF) chromatin remodeling complex. It is essential for controlling gene expression, primarily by altering chromatin architecture via the energy derived from ATP breakdown (Carey et al., 2015; Zhou S. et al., 2021). Consequently, genes regulated by BRG1 are critically involved in diverse biological processes and diseases, including cancer (Medina and Sanchez-Cespedes, 2008), liver regeneration (Fan et al., 2022), intestinal immunity (Qi et al., 2021) and heart disease (Li et al., 2019). In the cardiovascular system, BRG1 is indispensable for maintaining mesodermal identity and ensuring proper cardiac development (Hota et al., 2022). Beyond development, BRG1 also participates in various acquired cardiac pathologies. For instance, during pathological cardiac hypertrophy, both BRG1 and the microphthalmia-associated transcription factor (MITF) are upregulated and cooperatively regulate the expression of the pro-hypertrophic transcription factor GATA4 (Mehta et al., 2015). In models of myocardial ischemia-reperfusion injury, endothelial BRG1 also exacerbates myocardial injury by interacting with JMJD2B to promote the transactivation of the neutrophil ligand PODXL, thereby enhancing neutrophil infiltration (Zhang et al., 2018). Notably, BRG1 has been identified as a pro-fibrotic driver in multiple extracardiac tissues (Zhou Y. et al., 2021; Jolly et al., 2023; Zhu et al., 2023). However, the function in myocardial fibrosis remains poorly understood.

ZEB1, also known as zinc finger E-box binding homeobox 1, plays a vital role in the ZEB family of transcription factors. It serves an essential function in epithelial-mesenchymal transition (EMT) (Zhang et al., 2015), being a primary regulator during embryonic development and cellular differentiation (Vandewalle et al., 2009). Furthermore, ZEB1 binds to various intracellular signaling molecules and proteins, forming complexes that regulate multiple signaling pathways, including Wnt/β-catenin, Hippo/YAP, and TGF-β/Smad3, thereby influencing biological processes such as fibrosis, tumor metastasis, and proliferation (Takeyama et al., 2010; Zhang et al., 2019; Feldker et al., 2020). It has been reported that miR-200b/c protects against LPS-induced early pulmonary fibrosis by targeting ZEB1/2 and inhibiting the TGF-β/Smad3 signaling pathway (Cao et al., 2018).

The TGF-β/Smad3 pathway is a well-established driver of fibrosis, with the phosphorylation status of Smad3 being dynamically regulated by a balance of kinases and phosphatases (Feng and Derynck, 2005). As a major serine/threonine phosphatase of the PPP family, PP2A serves as a critical negative regulator of numerous kinases and cellular functions (Chen et al., 2021). Notably, beyond its role in kinase inhibition, PP2A activity itself can be subject to upstream regulation. For instance, in the context of cancer, ZEB1 was shown to inhibit PP2A activity, thereby reducing the nuclear accumulation of HDAC4, which in turn promotes collagen I secretion and enhances tumor cell adhesion (Tan et al., 2022). This precedent positions PP2A as a potential key node in the fibrotic signaling network.

In our research, we found that BRG1 is a pivotal factor in driving collagen production, CFs proliferation, and fibrotic tissue formation by interacting with ZEB1 to negatively regulate the phosphatase PP2A, thereby increasing the phosphorylation and nuclear translocation of Smad3. These results indicate that BRG1 might serve as an effective therapeutic target for treating cardiac fibrosis.

## 2 Materials and methods

### 2.1 Animals

Healthy neonates from Kunming Mice (3 days postnatal) were purchased from the Experimental Animal Center of the Second Affiliated Hospital of Harbin Medical University. Male C57BL/6 mice (body weight 22–25 g, 8 weeks old) were obtained from Liaoning Changsheng Biotechnology Co., Ltd. All animals were kept in a temperature-controlled facility on a standard 12-h light/dark cycle, with ambient temperature maintained at 23 °C ± 3 °C and relative humidity at 30%–70%. All animal procedures were performed in accordance with the National Institutes of Health Guide for the Care and Use of Laboratory Animals and were approved under the institutional Animal Use License (No. SYXK(HEI)2022-022).

### 2.2 Adeno-associated virus serotype 9 (AAV9) infection and mouse model of MI

Intraperitoneal injections of 2,2,2-tribromoethanol (Sigma, United States) were administered to male C57BL/6 mice to induce anesthesia. After stabilization of vital signs and disappearance of the blink reflex, the chest area was shaved, and intubation was performed under a light source, followed by mechanical ventilation. The left anterior descending (LAD) coronary artery was tied off using 7/0 nylon sutures approximately 2–3 mm below the base of the left sinoatrial node to induce MI. Once the procedure was successfully completed, the chest was carefully closed, and both the muscle and skin layers were stitched. Each mouse was injected with 7.5 × 10^10^ viral genomes via the tail vein and administered lentiviral vectors carrying either AAV9-BRG1-shRNA or AAV9-NC-shRNA (FluidicLab, China). All the mice were maintained under identical feeding conditions, and samples were collected after 4 weeks.

### 2.3 Cell isolation and transfection

Neonatal mouse cardiac fibroblasts (CFs) were isolated from postnatal mice using a standard isolation protocol. First, the heart tissue was digested overnight with 0.25% trypsin (Solarbio, China), followed by enzymatic treatment with Type II collagenase (Invitrogen, United States). Following centrifugation at 1,500 rpm for 7 min, the cell pellet was resuspended in DMEM (Biosharp, China) and incubated for 90 min. Due to their natural adhesion properties, the fibroblasts settled onto the culture dish surface. The isolated CFs were cultured in DMEM under standard conditions (37 °C, 5% CO_2_, and 95% humidity in a humidified incubator).

Human CFs were obtained from ScienCell Research Laboratories (United States) and maintained in complete Fibroblast Medium (ScienCell, #2301) according to the supplier’s instructions. HEK-293 cells (Procell, China, Cat# CL-0001) were cultured in DMEM supplemented with 10% fetal bovine serum (FBS). The human CFs and HEK-293 cell line used in this study were not formally authenticated. Human CFs are primary cells obtained from a characterized commercial source (ScienCell) and were used at low passages. The HEK-293 cell line was also used at low passages following receipt from the supplier (Procell). All cell lines were kept at 37 °C in a 5% CO_2_ humidified incubator, and routinely tested for mycoplasma contamination.

For transfection, cells were transfected with Lipofectamine 2000™ (Invitrogen, United States) following the manufacturer’s instructions. The transfected materials included a BRG1 overexpression plasmid (Genechem, China) or small interfering RNAs (siRNAs) targeting mouse/human BRG1, ZEB1, or *Ppp2r1a*. All siRNAs and a scrambled negative control (siNC) were designed and synthesized by RiboBio (Guangzhou, China). The sense strand sequences are listed in Supplementary Table S2.

### 2.4 Quantitative real-time RT-PCR

Total RNA was extracted from cultured CFs or heart tissues dissected from the infarct zone using TRIzol™ Reagent (Invitrogen, United States) according to the manufacturer’s instructions. Quantitative real-time PCR was performed using SYBR Green Master Mix on an ABI 7500 Fast Real-Time PCR System (Applied Biosystems, United States). To ensure accurate quantification, we normalized the results relative to GAPDH as the internal control. The primer sequences utilized in this investigation are listed in Supplementary Table S1. The data were analyzed via the 2^−ΔΔCT^ method.

### 2.5 Wound healing assay

Prior to cell seeding, two horizontal guides were marked on the six-well plate for alignment. Cultured cells were scratched along the lines via a pipette tip and a ruler, then cleaned with PBS solution. The plate was divided into nine sections, which were aligned with the lines. Cells were subjected to transfection, and their healing process was monitored via a Nikon Ts100 microscope (Nikon, Tokyo, Japan) at 0 and 24 h.

### 2.6 Western blot

Protein samples were prepared from cultured CFs or cardiac tissues from the infarct zone using RIPA lysis buffer (Beyotime, China). Protein concentrations were determined by a BCA assay (Beyotime, China). Equal amounts of protein were then separated on 7.5%–12% SDS-PAGE gels and subsequently transferred onto nitrocellulose membranes (Millipore, United States). Following a quick 10 min blocking step with rapid blocking solution (GenScript, United States), the membranes were probed overnight at 4 °C with specific primary antibodies. To ensure specificity, a tailored incubation strategy was employed: for well-separated high-molecular-weight targets (BRG1, FN1, and Collagen I), the same membrane was sequentially incubated with rabbit anti-BRG1 (Sigma-Aldrich, United States, 1:500), anti-Collagen I (Proteintech, China, 1:1,000), and anti-FN1 (Proteintech, China, 1:1,000), each followed by fluorescent secondary antibody application. For absolute specificity, ZEB1 (Wanleibio, China, 1:1,000), Ppp2r1a (ABclonal, China, 1:1,000), phospho-Smad3 (Affinity, China, 1:500), and total Smad3 (Affinity, China, 1:500) were probed on separate membranes. The loading control, β-actin (Proteintech, China, 1:1,000), was detected on a parallel blot. After incubation with primary antibodies, membranes were washed with PBST (0.05% Tween-20 in PBS) and incubated for 1 h at room temperature in the dark with the appropriate fluorescent secondary antibodies (DyLight® 800 Goat Anti-Rabbit IgG or DyLight® 800 Goat Anti-Mouse IgG, 1:10,000, Abbkine, China). Protein bands were visualized using an ODYSSEY imaging system (LI-COR, United States), and band intensities were quantified with ImageJ software (NIH, United States) after normalization to β-actin.

### 2.7 Co-immunoprecipitation (Co-IP)

Co-IP assays were performed to investigate protein-protein interactions. Cells were lysed on ice with IP lysis buffer (Beyotime, China) containing protease inhibitors. After centrifugation at 12,000 × g for 15 min at 4 °C, the supernatants were collected and protein concentrations were determined. Equal amounts of protein lysates were incubated overnight at 4 °C with specific primary antibodies or control rabbit IgG (Cell Signaling Technology, China). Protein A/G magnetic beads (MedChemExpress, United States) were then added and incubated for 2 h at 4 °C. The beads were washed three times with cold PBST buffer, and bound proteins were eluted by boiling in 2× SDS loading buffer for 10 min. The immunoprecipitated proteins were subsequently analyzed by Western blot as described above.

### 2.8 EdU fluorescence staining

To evaluate DNA synthesis in CFs, we employed the Cell-Light EdU Apollo 567 In Vitro Kit (RiboBio, Guangzhou, China), following the manufacturer’s standard procedure. Briefly, CFs were exposed to 10 μM EdU in culture medium. After incubation, the cells were fixed using a 4% paraformaldehyde solution. To allow dye entry, cell membranes were permeabilized with 0.5% Triton X-100 (Biotopped, Beijing, China). Subsequently, 150 μL of Apollo staining reagent was added to each well and left to develop. The nuclei were then counterstained with DAPI (Beyotime, Shanghai, China). The stained samples were finally visualized under a fluorescence microscope (Carl Zeiss, Germany) to analyze the results.

### 2.9 Immunofluorescence staining

After transfection and treatment, the cells were subjected to fixation with 4% paraformaldehyde, followed by permeabilization with 0.4% Triton X-100 for 1 h. Subsequently, they were left to incubate with antibodies specific for BRG1 (Proteintech, China, 1:100), Smad3 (Affinity, China, 1:300), and ZEB1 (Invitrogen, United States, 1:1,000). Following three thorough PBS washes, the cells were treated with either fluorescein-488 or fluorescein-594 antibodies. Nuclei were stained with DAPI in the dark. Finally, the samples were observed via a fluorescence microscope (Carl Zeiss, Germany).

### 2.10 Echocardiographic measurements

Four weeks following MI, cardiac function was assessed using transthoracic echocardiography with the Vevo 2100 High-Resolution Imaging System (Visual Sonics, Toronto, Canada). Key parameters, such as the left ventricular ejection fraction (EF), fractional shortening (FS), left ventricular internal diameter during diastole (LVIDd), and left ventricular internal diameter during systole (LVIDs), were measured to assess cardiac performance.

### 2.11 Masson’s trichrome staining

Four weeks following MI, we harvested the hearts, soaked them in 4% paraformaldehyde for a full day, and then got them ready for embedding in paraffin. The tissues were sectioned to a thickness of approximately 5 μm. After dewaxing and rehydrating, the sections were stained with Masson’s Trichrome Staining Kit (Solarbio, Beijing, China). The extent of collagen accumulation was quantified using Image-Pro Plus software, with the proportion of scar tissue calculated by measuring the blue-stained collagen regions in the stained sections.

### 2.12 Chromatin immunoprecipitation (ChIP) assay

ChIP assays were performed via a ChIP assay kit (Thermo Scientific, MA, United States). First, CFs underwent formaldehyde crosslinking, followed by cell lysis and DNA shearing. Antibodies specific to BRG1 (Proteintech, China), ZEB1 (Invitrogen, United States), or rabbit IgG (Cell Signaling Technology, Danvers, MA) were used to pull down the protein-DNA complexes. The crosslinks were then broken, and the DNA was isolated for downstream analysis.

### 2.13 Luciferase reporter assays

The *Ppp2r1a* promoter reporter plasmid was constructed by cloning a 1.5-kb fragment of the mouse *Ppp2r1a* promoter region into the pGL4.10[luc2] vector (Promega, United States). HEK-293 cells were seeded in 24-well plates and grown to 90% confluence before transfection. Cells were co-transfected with the *Ppp2r1a* reporter construct along with either SMARCA4 overexpression plasmid, ZEB1-targeting siRNA, or BRG1-targeting siRNA using Lipofectamine 2000 transfection reagent in Opti-MEM medium (Invitrogen, United States) according to the manufacturer’s protocol. Forty-eight hours after transfection, cell lysates were prepared and luciferase activity was measured using the Dual-Luciferase Reporter Assay System (Promega, United States) following the manufacturer’s instructions. Firefly luciferase activity was normalized to Renilla luciferase activity for data analysis.

### 2.14 Cell counting kit-8 (CCK-8) assay

Cell viability was assessed using the CCK-8 (Beyotime, China) according to the manufacturer’s instructions. In brief, CFs were seeded in 96-well plates at a density of 5 × 10^3^ cells per well in complete growth medium and allowed to adhere overnight. After varying treatments for 24 h, 10 µL of CCK-8 reagent was directly added to each well, followed by incubation at 37 °C for 4–6 h. The absorbance of each well at 450 nm was measured using a microplate reader (BioTek, Richmond,·United States).

### 2.15 PP2A activity assay

PP2A phosphatase activity in CFs was measured using the Malachite Green Phosphate Detection Kit (Beyotime, China) according to the manufacturer’s instructions. Following treatments, cells were lysed and centrifuged at 12,000 × g for 15 min at 4 °C. Total protein concentration was determined by BCA assay. Equal amounts of protein lysate were incubated with a PP2A-specific phosphopeptide substrate for 30 min at 37 °C. The reaction was terminated by adding malachite green reagent, and absorbance was measured at 630 nm after 30 min incubation. PP2A activity was calculated using a phosphate standard curve and normalized to total protein content.

### 2.16 Statistical analysis

All statistical tests were analyzed using GraphPad Prism version 9.5 (GraphPad Software) and the data in this study are shown as mean ± S.E.M. For comparisons between two groups, a *t*-test was employed. When evaluating differences across several groups, a one-way ANOVA was conducted, followed by *post hoc* tests such as Tukey’s adjustments to pinpoint specific contrasts. For experiments involving two independent variables, two-way ANOVA was performed, followed by Tukey’s *post hoc* test for multiple comparisons among all experimental groups. *p* < 0.05 was considered statistically significant.

## 3 Results

### 3.1 BRG1 expression is elevated during cardiac fibrosis

**FIGURE 1:**
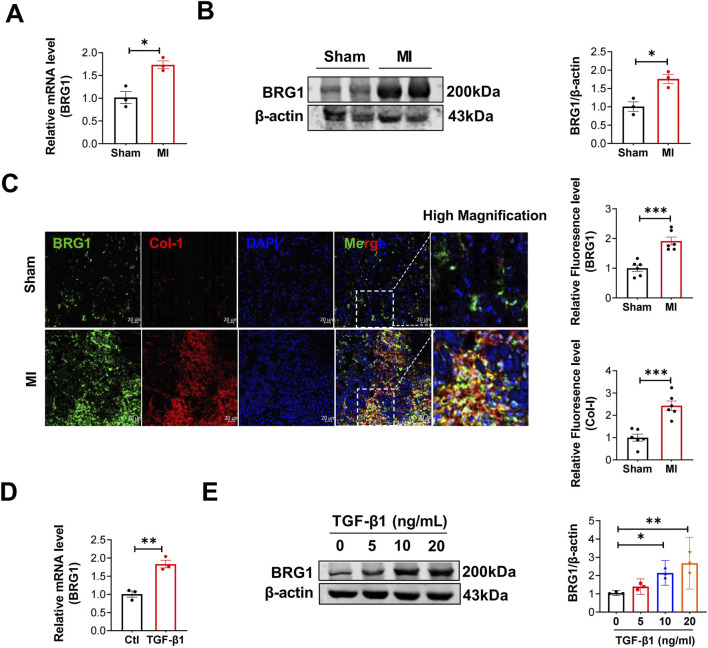
BRG1 was upregulated in mice after 4 weeks of MI and in CFs exposed to TGF-β1. **(A,B)** Levels of BRG1 mRNA and protein in the infarcted mouse hearts after MI for 4 weeks. *p < 0.05 vs. sham group by a two-tailed Student’s t-test. n = 3. **(C)** Immunofluorescence staining of BRG1 in the infarcted mouse hearts after MI for 4 weeks. BRG1 (green), Col-I (red) and nuclei (DAPI, blue) were labeled. Scale bar = 20 μm ***p < 0.001 vs. sham group by a two-tailed Student’s t-test. n = 6. **(D)** mRNA level of BRG1 in cultured CFs exposed to TGF-β1 (10 ng/mL). **p < 0.01 vs. Ctl group by a two-tailed Student’s t-test. n = 3. **(E)** Protein level of BRG1 in cultured CFs exposed to TGF-β1 dose-dependently (0, 5, 10, 20 ng/mL). *p < 0.05, **p < 0.01 vs. 0 ng/mL group by one-way ANOVA followed by Tukey’s *post hoc* analysis. n = 3.

To investigate the function of BRG1 in cardiac fibrosis, we initially assessed its expression in MI models and in cultured CFs exposed to TGF-β1. Our previous study demonstrated a steady increase in BRG1 expression in the border zone following MI, reaching its highest point by day 7 and maintaining elevated levels through days 14 and 30 (Li et al., 2023). Four weeks after MI, the elevation of BRG1 in the infarct zone of mouse hearts was confirmed by both mRNA and protein assays (Figures 1A,B). The immunofluorescence staining further showed that BRG1 expression is increased in the infarct zone of mouse hearts compared to sham hearts (Figure 1C). Similarly, in cultured CFs exposed to 20 ng/mL TGF-β1 for 24 h, BRG1 mRNA levels were elevated relative to those in the control group (Figure 1D). Additionally, TGF-β1 significantly boosted BRG1 protein levels in a manner that was directly proportional to the dose administered (Figure 1E).

### 3.2 BRG1 knockdown attenuates cardiac fibrosis after MI

**FIGURE 2:**
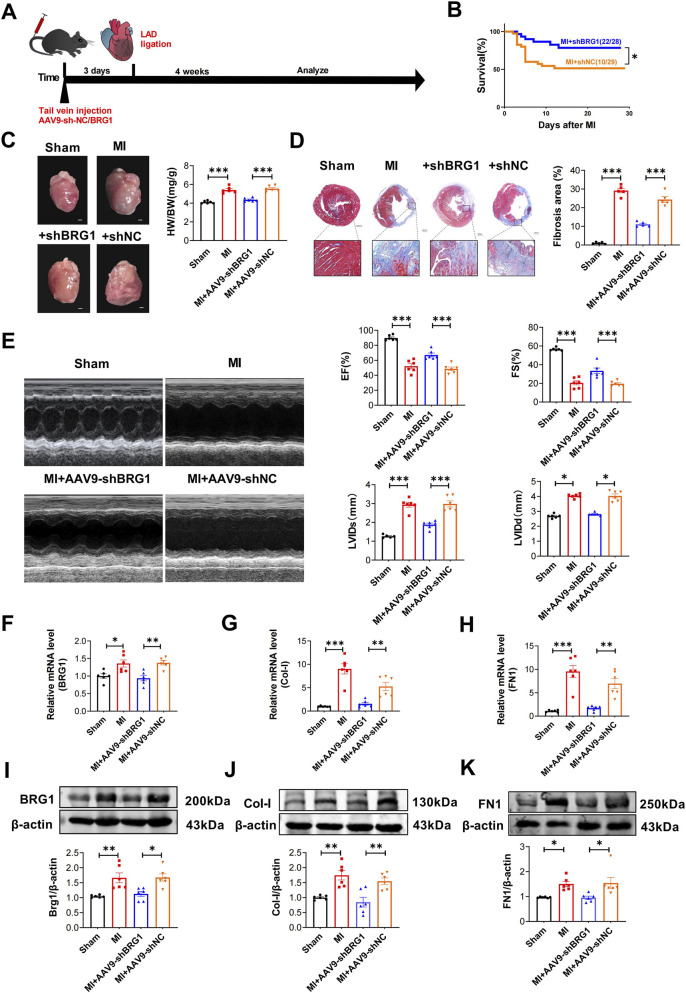
BRG1 knockdown in mice prevented cardiac fibrosis after MI. **(A)** Schematic diagram of the experiment. Tail vein injection of AAV9-BRG1-shRNA or AAV9-NC-shRNA. **(B)** Kaplan–Meier survival curves. AAV9-BRG1-shRNA and AAV9-NC-shRNA mice survival were recorded within 4 weeks post-MI. *p < 0.05 vs. MI+AAV9-shNC group by log-rank test. n = 28–29. **(C)** Cardiac morphology and heart weight/body weight (HW/BW) ratio analysis across experimental groups. ***p < 0.001 vs. Sham group or MI+AAV9-shNC group by one-way ANOVA followed by Tukey’s *post hoc* test. n = 6. Scale bar = 1 mm. **(D)** Masson’s trichrome-stained coronal heart sections showing myocardial architecture and border zone fibrosis. Fibrotic area quantification (infarct size) was performed using Image-Pro Plus software. ***p < 0.001 vs. Sham group or MI+AAV9-shNC group by one-way ANOVA followed by Tukey’s *post hoc* test. n = 5. Scale bar = 50 μm. **(E)** Echocardiographic measurement of cardiac function. EF, ejection fraction; FS, fractional shortening; LVIDd, left ventricular internal diameter in diastole; LVIDs, left ventricular internal diameter in systole. *p < 0.05, ***p < 0.001 vs. Sham group or MI+AAV9-shNC group by one-way ANOVA followed by Tukey’s *post hoc* analysis. n = 6. **(F–H)** mRNA levels of BRG1, Col-I and FN1. *p < 0.05, **p < 0.01, ***p < 0.001 vs. Sham group or MI+AAV9-shNC group by one-way ANOVA followed by Tukey’s *post hoc* test. n = 6. **(I–K)** Protein expression levels of BRG1, Col-I and FN1. *p < 0.05, **p < 0.01 vs. Sham group or MI+AAV9-shNC group by one-way ANOVA followed by Tukey’s *post hoc* test. n = 6.

Next, we explored how BRG1 influences cardiac fibrosis in mouse MI models. We delivered AAV9 vectors carrying either BRG1-targeted shRNA or non-specific control shRNA via tail vein injections. Three days postinjection, we induced MI through coronary artery ligation, and 4 weeks down the line, we evaluated the extent of interstitial fibrosis (Figure 2A). As shown in Figure 2B, BRG1 knockdown decreased the mortality after MI. Furthermore, 4 weeks following MI, BRG1 knockdown reduced the heart weight-to-body weight (HW/BW) ratio and infarct area (Figures 2C,D). Echocardiographic analysis demonstrated that BRG1 knockdown improved cardiac function in 4-week MI model mice, as evidenced by increased ejection fraction (EF) and fractional shortening (FS), as well as decreased left ventricular internal diameters in diastole (LVIDd) and systole (LVIDs) (Figure 2E). As expected, BRG1 knockdown also reversed the MI-induced upregulation of BRG1, Col-I, and FN1 at both the mRNA and protein levels (Figures 2F–K). These findings demonstrated that suppressing BRG1 enhanced cardiac function and inhibited harmful cardiac remodeling after MI.

### 3.3 BRG1 regulates collagen production and proliferation of CFs *in vitro*

**FIGURE 3:**
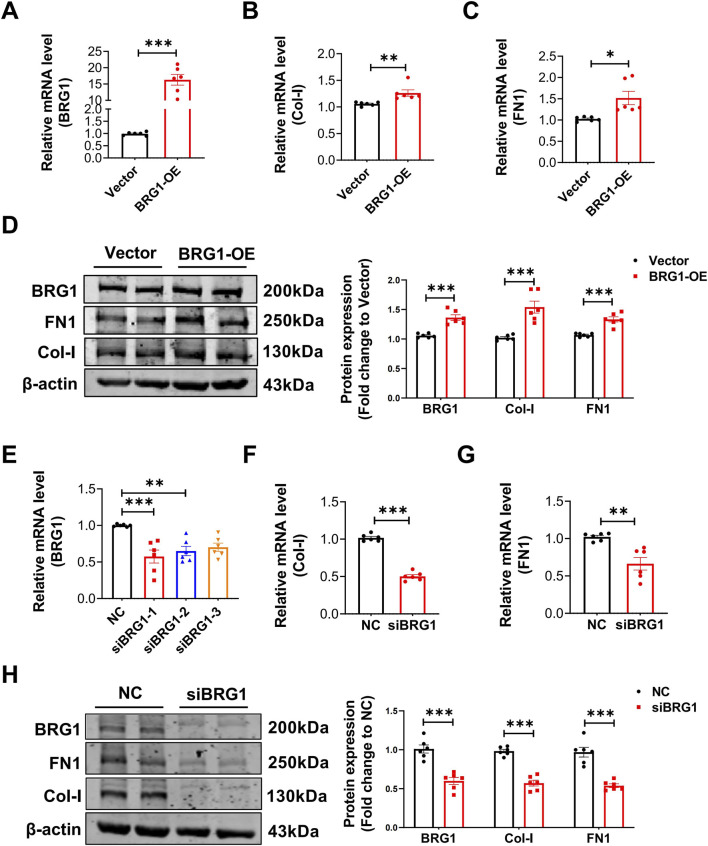
The effect of BRG1 overexpression or knockdown on collagen synthesis in CFs. **(A)** BRG1 overexpression efficiency was detected by qRT-PCR. ***p < 0.001 vs. Vector group by a two-tailed Student’s t-test. n = 6. **(B,C)** mRNA levels of Col-I and FN1 in CFs following BRG1 overexpression. *p < 0.05, **p < 0.01 vs. Vector group by a two-tailed Student’s t-test. n = 6. **(D)** Protein levels of Col-I and FN1 in CFs following BRG1 overexpression. ***p < 0.001 vs. Vector group by a two-tailed Student’s t-test. n = 6. **(E)** Transfection efficiency of BRG1 siRNAs was detected by qRT-PCR. **p < 0.01, ***p < 0.001 vs. NC group by one-way ANOVA followed by Tukey’s *post hoc* analysis. n = 6. **(F,G)** mRNA levels of Col-I and FN1 in CFs following BRG1 knockdown. **p < 0.01, ***p < 0.001 vs. NC group by a two-tailed Student’s t-test. n = 6. **(H)** Protein expression levels of Col-I and FN1 in CFs following BRG1 knockdown. ***p < 0.001 vs. NC group by two-tailed Student’s t-test. n = 6.

To determine the effect of BRG1 on CFs, we conducted overexpression and knockdown experiments using BRG1 plasmids and siRNAs. As depicted in Figure 3A, successful BRG1 overexpression was confirmed in CFs transfected with the overexpression plasmids. Exogenous BRG1 overexpression significantly increased the mRNA and protein levels of Col-I and FN1 (Figures 3B–D). These results showed that BRG1 overexpression enhances collagen synthesis in CFs. Conversely, as shown in Figure 3E, three siRNAs targeting BRG1 were designed, with siBRG1-1 demonstrating the highest silencing efficiency. The knockdown of endogenous BRG1 by siBRG1 inhibited the mRNA and protein expression of Col-I and FN1 (Figures 3F–H).

**FIGURE 4:**
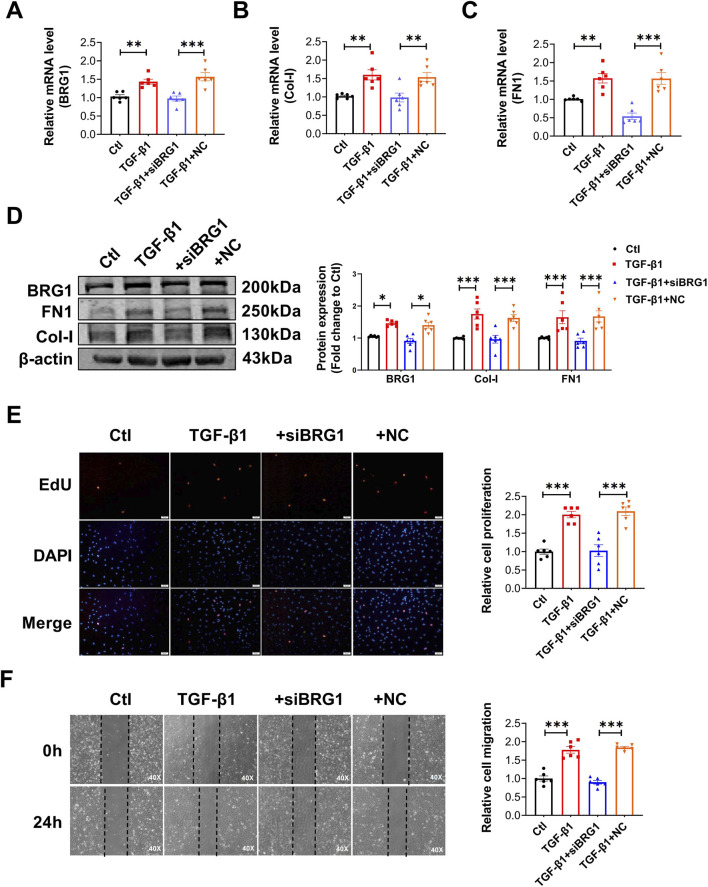
BRG1 knockdown attenuated TGF-β1-induced profibrotic responses in CFs, including collagen synthesis, proliferation and migration. **(A–C)** mRNA levels of BRG1, Col-I and FN1 in CFs treated with TGF-β1 and transfected with siBRG1. **p < 0.01, ***p < 0.001 vs. Ctl group or TGF-β1+NC group by one-way ANOVA followed by Tukey’s *post hoc* analysis. n = 6. **(D)** Protein levels of BRG1, Col-I and FN1 in CFs treated with TGF-β1 and transfected with siBRG1. *p < 0.05, ***p < 0.001 vs. Ctl group or TGF-β1+NC group by two-way ANOVA followed by Tukey’s *post hoc* analysis. n = 6. **(E)** EdU fluorescence staining of CFs proliferation following TGF-β1 stimulation and BRG1 knockdown. Scale bar = 50 μm ***p < 0.001 vs. Ctl group or TGF-β1+NC group by one-way ANOVA followed by Tukey’s *post hoc* analysis. n = 6. **(F)** Wound healing assessing CFs migration under TGF-β1 stimulation and BRG1 knockdown. Scale bar = 100 μm ***p < 0.001 vs. Ctl group or TGF-β1+NC group by one-way ANOVA followed by Tukey’s *post hoc* analysis. n = 6.

To further investigate the regulatory role of BRG1, we evaluated its influence on TGF-β1-driven activation of CFs. BRG1 knockdown decreased the TGF-β1-triggered upregulation of BRG1, Col-I, and FN1 in CFs at both the protein and mRNA levels (Figures 4A–D). EdU fluorescence staining revealed that BRG1 knockdown significantly reduced the proliferation of TGF-β1-stimulated fibroblasts (Figure 4E). Subsequently, a CCK-8 assay confirmed this effect by measuring a significant decrease in cell viability (Supplementary Figure S1). Moreover, the wound healing study indicated that the reduction of BRG1 inhibited effect on the migration of fibroblasts triggered by TGF-β1 (Figure 4F). These findings suggest that BRG1 plays a crucial role in modulating cardiac fibrosis at the cellular level.

### 3.4 Identification of ZEB1 as a functional partner of BRG1

**FIGURE 5:**
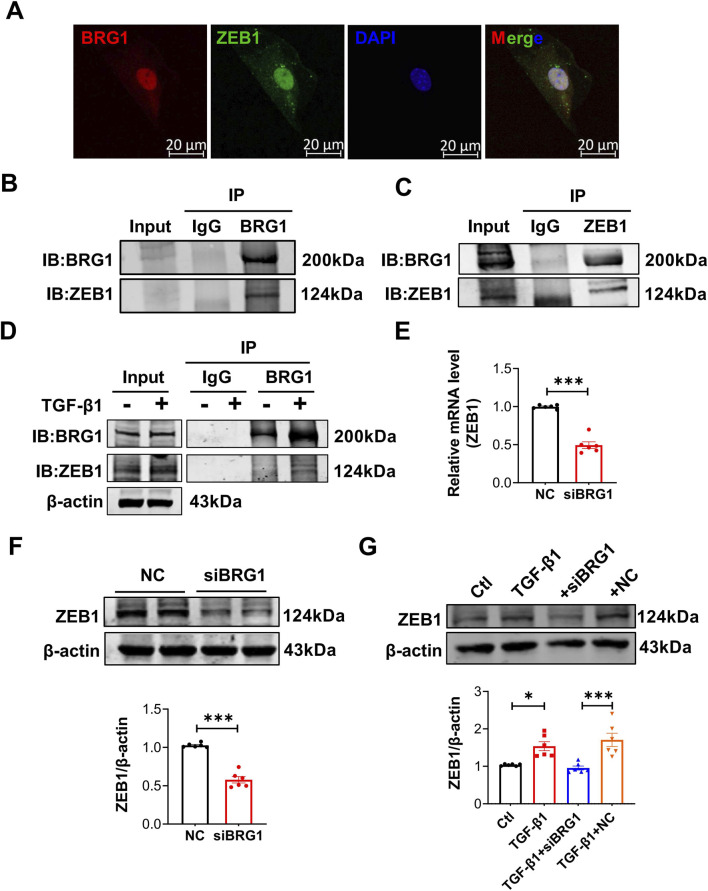
BRG1 interacts with ZEB1 and is required to maintain ZEB1 expression. **(A)** Immunofluorescence staining showing the cellular distribution of BRG1 and ZEB1 in CFs. BRG1 was stained in red, and ZEB1 was stained in green. Scale bar = 20 μm. **(B,C)** Protein-protein interaction between BRG1 and ZEB1 in CFs was validated by co-immunoprecipitation (IP) in CFs. **(B)** ZEB1 co-precipitated with anti-BRG1 antibody. **(C)** BRG1 co-precipitated with anti-ZEB1 antibody. **(D)** The interaction between BRG1 and ZEB1 in CFs treated with TGF-β1 (10 ng/mL) or untreated was confirmed by IP with anti-BRG1. **(E,F)** ZEB1 levels in CFs transfected with siBRG1 were quantified by qRT-PCR and Western blot. ***p < 0.001 vs. NC group by a two-tailed Student’s t-test. n = 6. **(G)** ZEB1 protein levels in CFs transfected with siBRG1 and treated with TGF-β1 *p < 0.05, ***p < 0.001 vs. Ctl group or TGF-β1+NC group by one-way ANOVA followed by Tukey’s *post hoc* analysis. n = 6.

BRG1, a key component of the chromatin remodeling complex, is crucial for regulating gene expression through interactions with various nuclear proteins (Bisht et al., 2022). To clarify the mechanism by which BRG1 influences cardiac fibrosis, we used the STRING database to predict BRG1-interacting proteins. The transcription factor ZEB1, a known mediator of fibrosis (Cheng et al., 2021), was identified as a candidate. This interaction is biologically plausible, as it has been previously demonstrated that ZEB1 can bind to BRG1 (Sanchez-Tillo et al., 2010). Immunofluorescence analysis revealed nucleolar colocalization of ZEB1 and BRG1 in CFs (Figure 5A), with reciprocal co-immunoprecipitation experiments confirming their physical interaction (Figures 5B,C). We further confirmed that TGF-β1 stimulation enhanced the interaction between endogenous BRG1 and ZEB1 (Figure 5D). Interestingly, we found that BRG1 is required to maintain ZEB1 protein levels, as BRG1 knockdown significantly decreased ZEB1 expression and suppressed its TGF-β1-induced upregulation in CFs (Figures 5E–G). These results confirm our hypothesis that BRG1 interacts with ZEB1 and regulates its protein expression in CFs.

### 3.5 ZEB1 mediates the role of BRG1 in the development of cardiac fibrosis

**FIGURE 6:**
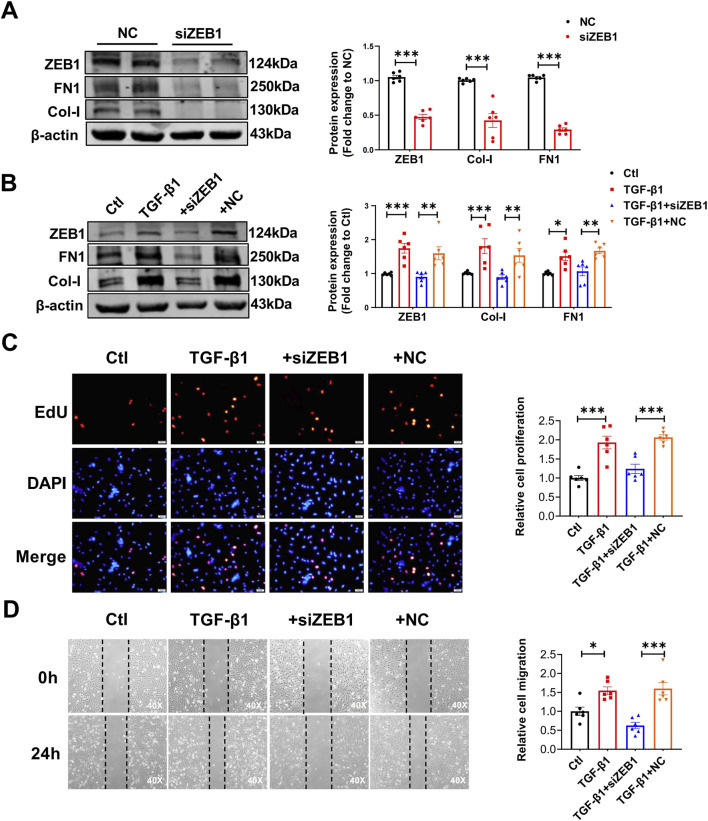
The effects of ZEB1 knockdown in CFs. **(A)** Western blot analysis of ZEB1, Col-I and FN1 protein expression following ZEB1 siRNAs transfection in CFs. ***p < 0.001 vs. NC group by a two-tailed Student’s t-test. n = 6. **(B)** Protein levels of ZEB1, Col-I, and FN1 in CFs transfected with siZEB1 and treated with TGF-β1. *p < 0.05, **p < 0.01 and ***p < 0.001 vs. Ctl group or TGF-β1+NC group by two-way ANOVA followed by Tukey’s *post hoc* analysis. n = 6. **(C)** EdU fluorescence assay assessing proliferation of CFs transfected with siZEB1 and treated with TGF-β1. Scale bar = 50 μm ***p < 0.001 vs. Ctl group or TGF-β1+NC group by one-way ANOVA followed by Tukey’s *post hoc* analysis. n = 6. **(D)** Wound healing assay assessing migration of CFs transfected with siZEB1 and treated with TGF-β1. Scale bar = 100 μm *p < 0.05, ***p < 0.001 vs. Ctl group or TGF-β1+NC group by one-way ANOVA followed by Tukey’s *post hoc* analysis. n = 6.

To evaluate whether ZEB1 participates in BRG1-induced CFs activation, we synthesized siRNAs targeting ZEB1 (siZEB1) and confirmed successful ZEB1 knockdown, which simultaneously reduced Col-I and FN1 protein levels (Figure 6A). Similarly, ZEB1 knockdown attenuated TGF-β1-induced fibrotic responses, including the upregulation of Col-I and FN1, cell proliferation, collagen synthesis and cell viability (Figures 6B–D; Supplementary Figure S2).

**FIGURE 7:**
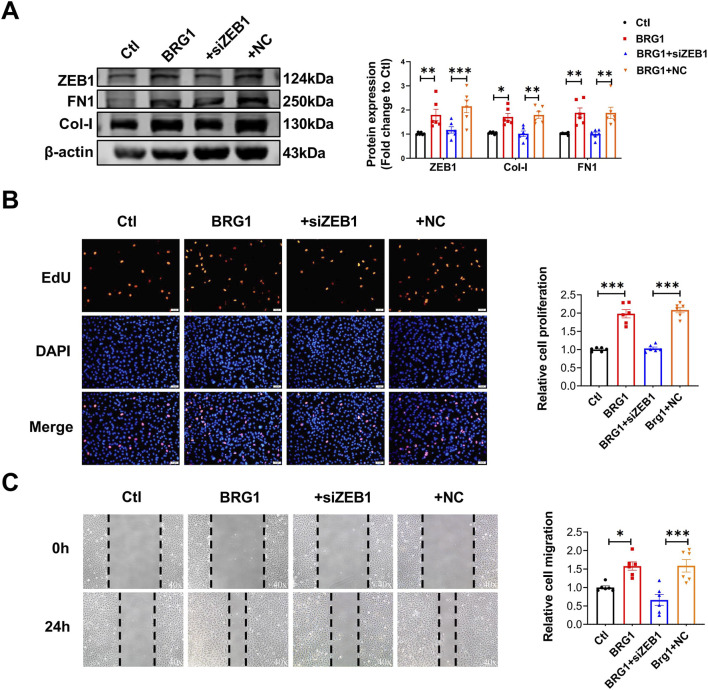
ZEB1 regulated the effects of BRG1 on cardiac fibrosis. **(A)** Western blot analysis of ZEB1, Col-I and FN1 protein levels in CFs following co-transfection with BRG1 overexpression plasmids and siZEB1. *p < 0.05, **p < 0.01, ***p < 0.001 vs. Ctl group or BRG1+NC group by two-way ANOVA followed by Tukey’s *post hoc* analysis. n = 6. **(B)** Proliferative activity of CFs co-transfected with BRG1 and siZEB1 was assessed by EdU staining. Scale bar = 50 μm ***p < 0.001 vs. Ctl group or BRG1+NC group by one-way ANOVA followed by Tukey’s *post hoc* analysis. n = 6. **(C)** Cell migration was evaluated in CFs co-transfectied with BRG1 and siZEB1 by wound healing. Scale bar = 100 μm *p < 0.05, ***p < 0.001 vs. Ctl group or BRG1+NC group by one-way ANOVA followed by Tukey’s *post hoc* analysis. n = 6.

Next, we explored whether ZEB1 plays a role in BRG1-driven activation of CFs. Our findings showed that ZEB1 knockdown reduced BRG1-induced collagen synthesis in CFs (Figure 7A). Additionally, the proliferative response, collagen production and cell viability triggered by BRG1 were effectively blocked when cells were simultaneously transfected with siZEB1 (Figures 7B,C; Supplementary Figure S3). These results indicate that ZEB1 plays a critical role in mediating the regulatory effects of BRG1 on the post-MI cardiac fibrosis process.

### 3.6 BRG1 promotes Smad3 phosphorylation by regulating *Ppp2r1a* transcription

**FIGURE 8:**
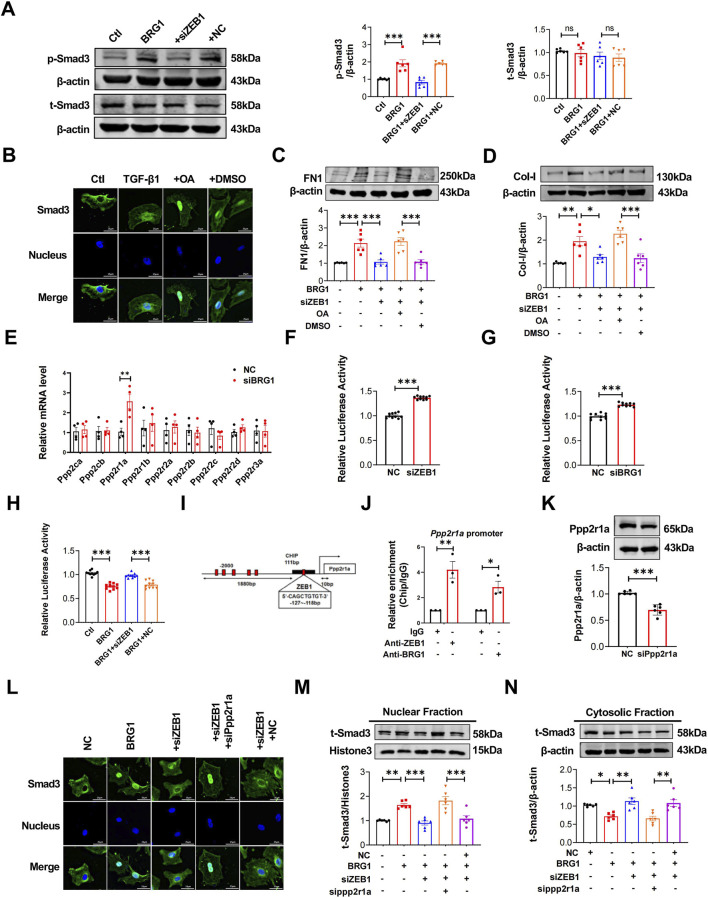
BRG1 promoted Smad3 phosphorylation by regulating *Ppp2r1a* transcription. **(A)** Western blot analysis of p-Smad3 and t-Smad3 protein levels in CFs following co-transfection with BRG1 and siZEB1. ***p < 0.001 vs. Ctl group or BRG1+NC group by one-way ANOVA followed by Tukey’s *post hoc* analysis. n = 6. **(B)** Immunofluorescence staining reveals the nuclear translocation of Smad3 in CFs. Smad3 (green) and nuclei (DAPI, blue) were labeled. Scale bar = 25 μm. **(C,D)** Western blot analysis of Col-I and FN1 protein levels in CFs treated with OA following co-transfection with BRG1 overexpression plasmids and siZEB1. *p < 0.05, **p < 0.01, ***p < 0.001 vs. indicated groups by one-way ANOVA followed by Tukey’s *post hoc* analysis. n = 6. **(E)** In primary mouse fibroblasts with siBRG1, qRT-PCR was employed to evaluate PP2A subunit (*Ppp2ca*, *Ppp2cb*, *Ppp2r1a*, *Ppp2r1b*, *Ppp2r2a*, *Ppp2r2b*, *Ppp2r2c*, *Ppp2r2d*, *Ppp2r3a*) mRNA expression. **p < 0.01 vs. NC group by a two-tailed Student’s t-test. n = 4. **(F)** Luciferase reporter assay of *Ppp2r1a* promoter activity in CFs transfected with siZEB1. ***p < 0.001 vs. NC group. **(G)** Luciferase reporter assay of *Ppp2r1a* promoter activity in CFs transfected with siBRG1. ***p < 0.001 vs. NC group. **(H)** Luciferase reporter assay of *Ppp2r1a* promoter activity in CFs co-transfected with BRG1 overexpression plasmid and siZEB1 ***p < 0.001 vs. Ctl group or BRG1+NC group by one-way ANOVA followed by Tukey’s *post hoc* analysis. n = 10. **(I)** ChIP analysis identified ZEB1 binding sites in the *Ppp2r1a* promoter region. **(J)** ChIP-qPCR analysis of BRG1 and ZEB1 binding to the *Ppp2r1a* promoter region in CFs. *p < 0.05 and **p < 0.01 vs. IgG. n = 3. **(K)** Transfection efficiency of siPpp2r1a was detected by Western blot, ***p < 0.001 vs. NC group by a two-tailed Student’s t-test. n = 6. **(L)** Immunofluorescence detects nuclear translocation of Smad3 (green) in CFs, with DAPI (blue) marking nuclei. Scale bar = 25 μm. **(M,N)** Representative Western blotting analysis of t-Smad3 protein level in cytoplasm and nucleus in CFs. β-actin was used as the loading control for cytosolic fractions. Histone3 was used as the loading control for nuclear fractions. *p < 0.05, **p < 0.01, ***p < 0.001 vs. indicated groups by one-way ANOVA followed by Tukey’s *post hoc* analysis. n = 6.

As Smad3 is a key driver of fibrosis (Xu et al., 2016), we sought to determine whether BRG1 engages in this classical signaling pathway through its interaction with ZEB1. Our results demonstrated that ZEB1 knockdown blocked BRG1-induced Smad3 phosphorylation (p-Smad3) without altering total Smad3 (t-Smad3) protein levels (Figure 8A), suggesting the BRG1/ZEB1 complex acts upstream of Smad3 activation. Based on the established role of PP2A in dephosphorylating and inactivating Smad3 (Rizvi et al., 2018), We hypothesized that the BRG1-ZEB1 complex might promote cardiac fibrosis by activating Smad3 through the inactivation of PP2A. Supporting this hypothesis, TGF-β1 stimulation was found to suppress PP2A activity by the Malachite Green Phosphate Detection Kit (Supplementary Figure S4). Importantly, BRG1 overexpression further enhanced this suppression, an effect that was largely reversed by ZEB1 knockdown (Supplementary Figure S5). Furthermore, treatment of CFs with the PP2A-specific inhibitor okadaic acid (OA, 10 nM) not only enhanced Smad3 nuclear accumulation (Figure 8B) but also rescued the reduction in collagen expression induced by BRG1/ZEB1 disruption (Figures 8C,D). These data strongly indicate that BRG1/ZEB1 turns off PP2A to activate Smad3, thereby promoting fibrosis.

We next explored how the BRG1/ZEB1 complex inactivates PP2A. To identify which PP2A subunit is involved, we screened their expression and found that *Ppp2r1a* (encoding the structural subunit Aα) as significantly upregulated upon BRG1 knockdown (Figure 8E). A dual luciferase reporter assay demonstrated that suppressing ZEB1 and BRG1 expression markedly enhanced *Ppp2r1a* promoter activity relative to that of the control (Figures 8F,G). Conversely, BRG1 overexpression strongly suppressed *Ppp2r1a* promoter activity, and this suppression was partially reversed by ZEB1 knockdown (Figure 8H). A Chromatin immunoprecipitation (ChIP) assays assay further revealed that ZEB1 binds to the *Ppp2r1a* promoter region (−121 to −10 bp) (Figures 8I,J). Western blot and qRT-PCR further verified that BRG1-mediated suppression of *Ppp2r1a* expression requires ZEB1 (Supplementary Figures S6, S7). To functionally validate *Ppp2r1a*’s role, we synthesized siRNAs targeting *Ppp2r1a* (si-*Ppp2r1a*) and confirmed successful *Ppp2r1a* knockdown (Figure 8K), which simultaneously elevated Col-I protein level (Supplementary Figure S8). Similarly, *Ppp2r1a* knockdown enhanced TGF-β1-induced fibrotic activation (Supplementary Figure S9). Finally, we confirmed that the impairment of Smad3 phosphorylation and nuclear translocation caused by ZEB1 knockdown could be rescued by concurrent *Ppp2r1a* knockdown, as evidenced by both immunofluorescence and cellular fractionation assays (Figures 8L–N).

Collectively, these findings demonstrate that the BRG1-ZEB1 complex transcriptionally represses *Ppp2r1a* to inhibit PP2A activity, thereby enhancing Smad3 phosphorylation and driving cardiac fibrosis.

### 3.7 BRG1 mediates pro-fibrotic activation in human CFs

**FIGURE 9:**
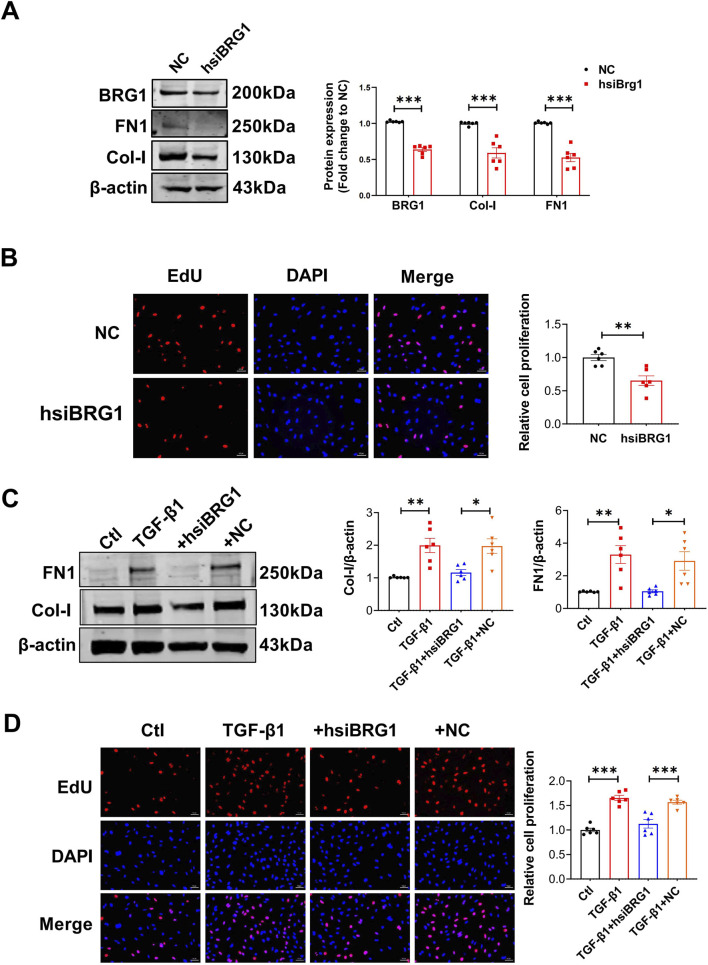
BRG1 silencing inhibited TGF-β1-induced activation of human CFs. **(A)** Western blot analysis of BRG1, Col-I and FN1 protein expression following hsiBRG1 transfection in human CFs. ***p < 0.001 vs. NC group by a two-tailed Student’s t-test. n = 6. **(B)** EdU fluorescence assay assessing proliferation of human CFs transfected with hsiBRG1. Scale bar = 50 μm **p < 0.01 vs. NC group by a two-tailed Student’s t-test. n = 6. **(C)** Protein levels of Col-I and FN1 in human CFs transfected with hsiBRG1 and treated with TGF-β1. *p < 0.05, **p < 0.01 vs. Ctl group or TGF-β1+NC group by one-way ANOVA followed by Tukey’s *post hoc* analysis. n = 6. **(D)** EdU fluorescence assay assessing proliferation of human CFs transfected with hsiBRG1 and treated with TGF-β1. Scale bar = 50 μm ***p < 0.001 vs. Ctl group or TGF-β1+NC group by one-way ANOVA followed by Tukey’s *post hoc* analysis. n = 6.

To validate the translational relevance of our findings, we investigated whether BRG1 plays a conserved role in human CFs. Knockdown of BRG1 using a human-specific siRNA (hsiBRG1) significantly reduced the protein levels of BRG1, Col-I, and FN1 under basal conditions (Figure 9A). BRG1 depletion also markedly suppressed human CFs proliferation (Figure 9B). Consistent with the results in mouse cells, BRG1 knockdown blunted the TGF-β1 induced upregulation of Col-I and FN1 (Figure 9C) and inhibited TGF-β1–stimulated fibroblast proliferation in human CFs (Figure 9D). These findings highlighted the potential of BRG1 as a therapeutic target in human fibrotic diseases.

## 4 Discussion

**FIGURE 10:**
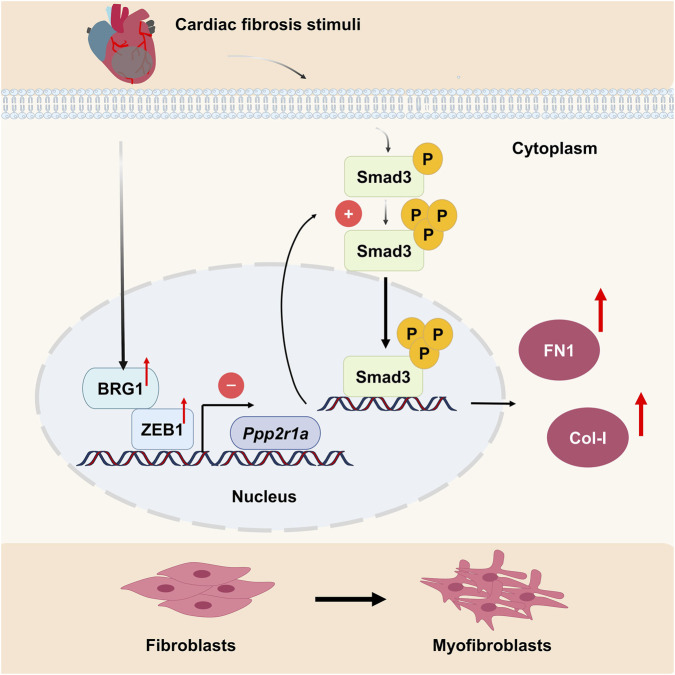
Schematic diagram depicting the role of BRG1 on cardiac fibrosis. Myocardial infarction upregulates BRG1, which interacts with ZEB1 to form a transcriptional repressor complex. This complex suppresses the expression of *Ppp2r1a* (encoding the PP2A structural subunit), leading to diminished PP2A activity. Consequently, Smad3 phosphorylation and nuclear translocation are enhanced, driving the transcription of fibrotic genes such as Col-1 and FN1, and ultimately exacerbating myocardial fibrosis.

Myocardial fibrosis is characterized by pathological remodeling, including cardiac myofibroblast proliferation and excessive collagen deposition, ultimately leading to heart dysfunction and failure (Liu et al., 2021). In this study, we identified a marked upregulation of BRG1 expression during fibrosis following MI. Functionally, BRG1 deficiency reduced collagen synthesis and myocardial fibrosis under both physiological and pathological conditions. Mechanistically, TGF-β1 enhanced the interaction between BRG1 and ZEB1 in CFs, forming a complex that transcriptionally repressed the structural subunit a of PP2A, encoded by *Ppp2r1a*. This suppression reduced PP2A activity, thereby enhancing Smad3 phosphorylation and nuclear translocation, and activating the TGF-β/Smad3 pathway to promote cardiac fibrosis. Our study suggests that BRG1 could be a potential target for treating myocardial fibrosis (Figure 10).

BRG1 is essential for modulating gene expression through chromatin regulation across different tissues and physiological states (Armstrong et al., 1998). In our prior investigation, tail vein injection of AAV9 carrying BRG1-specific shRNA effectively and selectively reduced BRG1 expression in the mouse heart, with no discernible impact on other major organs. In that study, BRG1 upregulation post-MI promoted arrhythmogenesis by modulating cardiac ion-channel transcription, and its knockdown reduced susceptibility to ventricular arrhythmias (Li et al., 2023). However, whether BRG1 is involved in post-MI myocardial fibrosis has remained unclear. Building on this, the present study provides the first systematic evidence that BRG1 acts as a direct driver of post-MI cardiac fibrosis, promoting cardiac fibroblast proliferation, enhancing collagen production, and accelerating fibrotic tissue deposition. This pro-fibrotic role of BRG1 is consistent with findings in other organ systems. Studies have shown that BRG1 also mediates hepatic stellate cell activation and renal interstitial fibrosis (Dong et al., 2020; Niu et al., 2022), suggesting that BRG1 may act as a central hub driving fibrosis in multiple organs. However, BRG1’s functions are highly context-dependent. In pressure overload-induced cardiac hypertrophy, the lncRNA Mhrt binds competitively to BRG1, inhibiting its chromatin remodeling activity and exerting protective effects (Han et al., 2014). This stands in sharp contrast to the fibrosis-promoting role of BRG1 observed in our MI model, highlighting the complexity of its regulatory networks in different pathological states. In summary, our findings establish BRG1 as a critical regulator of myocardial fibrosis.

In mammalian systems, the SWI/SNF chromatin remodeling complex serves as a master regulator of transcription initiation from chromatin templates and orchestrates fundamental cellular processes through dynamic interactions with lineage-specific transcriptional regulators (Armstrong et al., 1998). These interactions facilitate chromatin remodeling and transcription, which are essential for regulating gene expression. BRG1 contains an evolutionarily conserved ATPase catalytic domain, an N-terminal AT-hook bromodomain, and additional domains including QLQ, HSA, and BRK. The N-terminal and C-terminal domains of BRG1 are structurally and functionally essential for mediating its protein-protein interactions (Trotter and Archer, 2008). Previous studies show BRG1 interacts with zinc finger proteins to facilitate chromatin remodeling (Kadam and Emerson, 2003). In pulmonary fibrosis, IL-8 enhances the interaction between ZEB1 and the CD44/BRG1 complex, facilitating Sox2 promoter binding and contributing to fibrogenesis (Yang et al., 2021). Consistent with this, our study identifies ZEB1 as a key interaction partner of BRG1 in the heart, revealing a mechanism whereby BRG1 recruits ZEB1 to repress *Ppp2r1a* transcription, thereby promoting cardiac fibrosis via TGF-β/Smad3 signaling.

ZEB1, a core transcription factor of the zinc finger E-box binding homeobox family, regulates embryonic development and tissue homeostasis, and has been increasingly implicated in pathological processes including tumor metastasis, chemoresistance, and notably, fibrotic progression across multiple organs (Cheng et al., 2021). While ZEB1-BRG1 interaction has been documented in cancer and endothelial cells (Sanchez-Tillo et al., 2010), its role in cardiac fibrosis remained unclear. In the present study, we not only confirmed BRG1-ZEB1 physical interaction in CFs by Co-immunoprecipitation (Co-IP), but also demonstrated that this complex drives myocardial fibrosis through a previously unreported mechanism—transcriptional suppression of *Ppp2r1a*. Functionally, ZEB1 knockdown abolished TGF-β–induced fibroblast activation and suppressed collagen production. Moreover, ZEB1 deficiency reversed BRG1-induced Smad3 phosphorylation and myofibroblast transformation, indicating that ZEB1 is essential for BRG1-mediated pro-fibrotic signaling. Importantly, while BRG1 has been shown to transcriptionally regulate ZEB1 in other contexts, our data support a model in which ZEB1 serves as a critical molecular partner of BRG1 in the setting of cardiac fibrosis. Although ZEB1 has been linked to lung and kidney fibrosis, our work provides the first evidence of BRG1-ZEB1 cooperation in post-MI cardiac fibrosis.

The TGF-β/Smad3 signaling pathway is a well-established driver of fibrotic remodeling, exerting critical influence on post-MI myocardial fibrosis through transcriptional regulation of profibrotic genes (Liu and Feng, 2010). Tight control of TGF-β signaling is essential for cardiac homeostasis, with pathway activity being finely tuned not only by canonical post-translational modifications but also through reversible phosphorylation (Inoue and Imamura, 2008; Bengtsson et al., 2009). In this dynamic balance, kinase-mediated phosphorylation is counteracted by protein phosphatases that dephosphorylate signaling components and suppress pathway activation (Van Berlo et al., 2005). Among the diverse phosphatase families, the PPP subfamily—particularly PP2A—plays a prominent role in regulating TGF-β signaling (Petritsch et al., 2000; Batut et al., 2008). Notably, PP2A has been shown to directly dephosphorylate Smad3 under hypoxic conditions (Heikkinen et al., 2010).

Given that PP2A is a key negative regulator of Smad3 phosphorylation, we first investigated whether BRG1 modulates PP2A activity. Screening of all nine core subunits of the PP2A family revealed that knockdown of BRG1 specifically upregulated the mRNA level of *Ppp2r1a*, which encodes the structural subunit Aα. This result suggested the specific involvement of this subunit in BRG1-mediated fibrosis. Consistently, BRG1 overexpression suppressed *Ppp2r1a* promoter activity, an effect that was partially reversed by ZEB1 knockdown, while BRG1 or ZEB1 deletion enhanced *Ppp2r1a* transcription. Functionally, forced ZEB1 expression abolished the pro-fibrotic effects induced by BRG1, and PP2A inhibition mimicked BRG1 overexpression. Collectively, these data establish the suppression of PP2A as a key downstream event in BRG1-ZEB1 mediated fibrosis, which aligns with previous reports that deletion of *Ppp2r1a* promotes fibrotic progression in other tissues (Chen et al., 2021; Han et al., 2022).

While this study established the regulatory role of BRG1 in cardiac fibrosis, several limitations warrant consideration. First, although we identified the BRG1-ZEB1 complex as a transcriptional repressor of *Ppp2r1a*, the precise binding motif of this complex on the *Ppp2r1a* promoter requires further structural and molecular elucidation. Second, our findings would be strengthened by employing CF-specific BRG1 knockout or transgenic mouse models to definitively confirm its cell-autonomous profibrotic role and exclude potential contributions from other cell types. Third, our study focused on the TGF-β/Smad3 pathway, it remains unclear whether other key fibrogenic pathways [e.g., Wnt/β-catenin (Zhou Y. et al., 2021), Notch (Xiao et al., 2023)] interact with ZEB1 or BRG1, and thus their potential crosstalk in this context requires further study. Fourth, the protective effect of ZEB1 knockdown *in vivo* was not investigated in the current study. Moreover, our analysis was confined to a single 4- week post- MI time point, which precludes insights into the dynamics of BRG1 expression or its role in fibrosis resolution versus chronic progression. Finally, as our mechanistic insights derived mainly from murine models and *in vitro* studies using human CFs, their direct clinical relevance requires further validation in human failing heart tissues.

## 5 Conclusion

This study establishes BRG1 as a critical epigenetic regulator of post-MI myocardial fibrosis. Mechanistically, the BRG1-ZEB1 transcriptional complex drives fibrotic progression by suppressing *Ppp2r1a* expression, which enhances Smad3 phosphorylation and nuclear translocation. Our findings identify the BRG1-ZEB1-PP2A axis as a novel therapeutic target for attenuating cardiac fibrosis.

## Data Availability

The original contributions presented in the study are included in the article/Supplementary Material, further inquiries can be directed to the corresponding authors.
