# Supplementary material for: BRG1 exacerbates myocardial fibrosis after myocardial infarction by interacting with ZEB1
Source: Front Pharmacol. 2026 Mar 16;17:1802700. doi: 10.3389/fphar.2026.1802700 (PMC13033632; doi:10.3389/fphar.2026.1802700)
Supplement: Supplementary file 1 [file DataSheet1.docx]

**Supplemental information**

**Supplementary Figures**


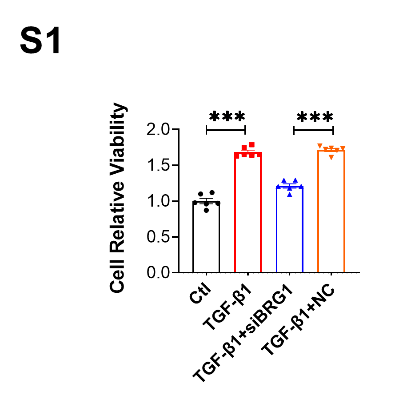


**Supplementary Figure 1. BRG1 knockdown attenuated TGF-β1-induced viability in CFs,** **related to Figure 4.** CCK-8 for the effects of siBRG1 on TGF-β1-induced viability of CFs. ****p* < 0.001 vs. Ctl group or TGF-β1+NC group by one-way ANOVA followed by Tukey’s post hoc analysis. n = 6.


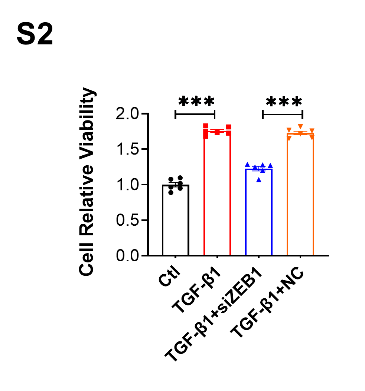


**Supplementary Figure 2. ZEB1 knockdown attenuated TGF-β1-induced viability in CFs, related to Figure 6.** CCK-8 for the effects of siZEB1 on TGF-β1-induced viability of CFs. ****p* < 0.001 vs. Ctl group or TGF-β1+NC group by one-way ANOVA followed by Tukey’s post hoc analysis. n = 6.


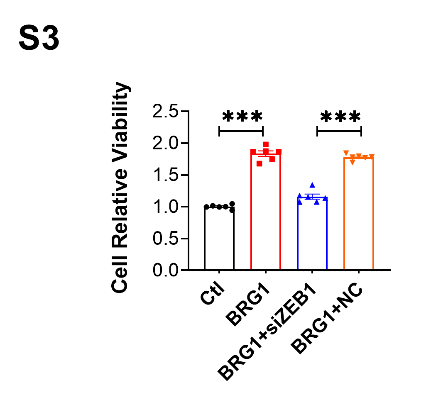


**Supplementary Figure 3. ZEB1 knockdown rescued BRG1-induced viability in CFs, related to Figure 7.** Cell viability was evaluated in CFs co-transfection with BRG1 and siZEB1 by CCK-8. ****p* < 0.001 vs. Ctl group or BRG1+NC group by one-way ANOVA followed by Tukey’s post hoc analysis. n = 6.


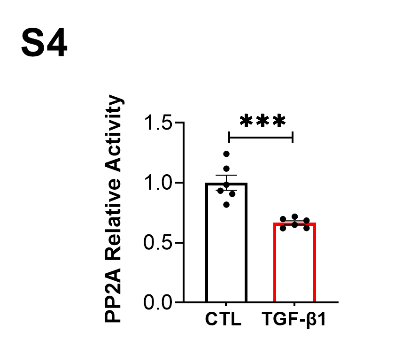


**Supplementary Figure 4. TGF-β1 suppressed PP2A activity in CFs, related to Figure 8.** PP2A activity was measured in CFs following TGF-β1 stimulation using the Malachite Green Phosphate Detection Kit. ****p* < 0.001 vs. Ctl group by a two-tailed Student’s *t*-test. n = 6.


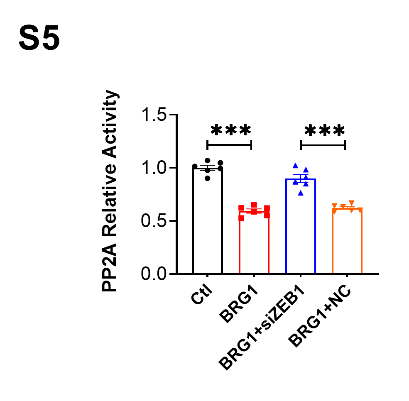


**Supplementary Figure 5. ZEB1 knockdown rescued BRG1-induced suppression of PP2A activity, related to Figure 8.** PP2A activity was measured in CFs following co-transfection with BRG1 overexpression plasmids and siZEB1 using the Malachite Green Phosphate Detection Kit. ****p* < 0.001 vs. Ctl group or BRG1+NC group by one-way ANOVA followed by Tukey’s post hoc analysis. n = 6.


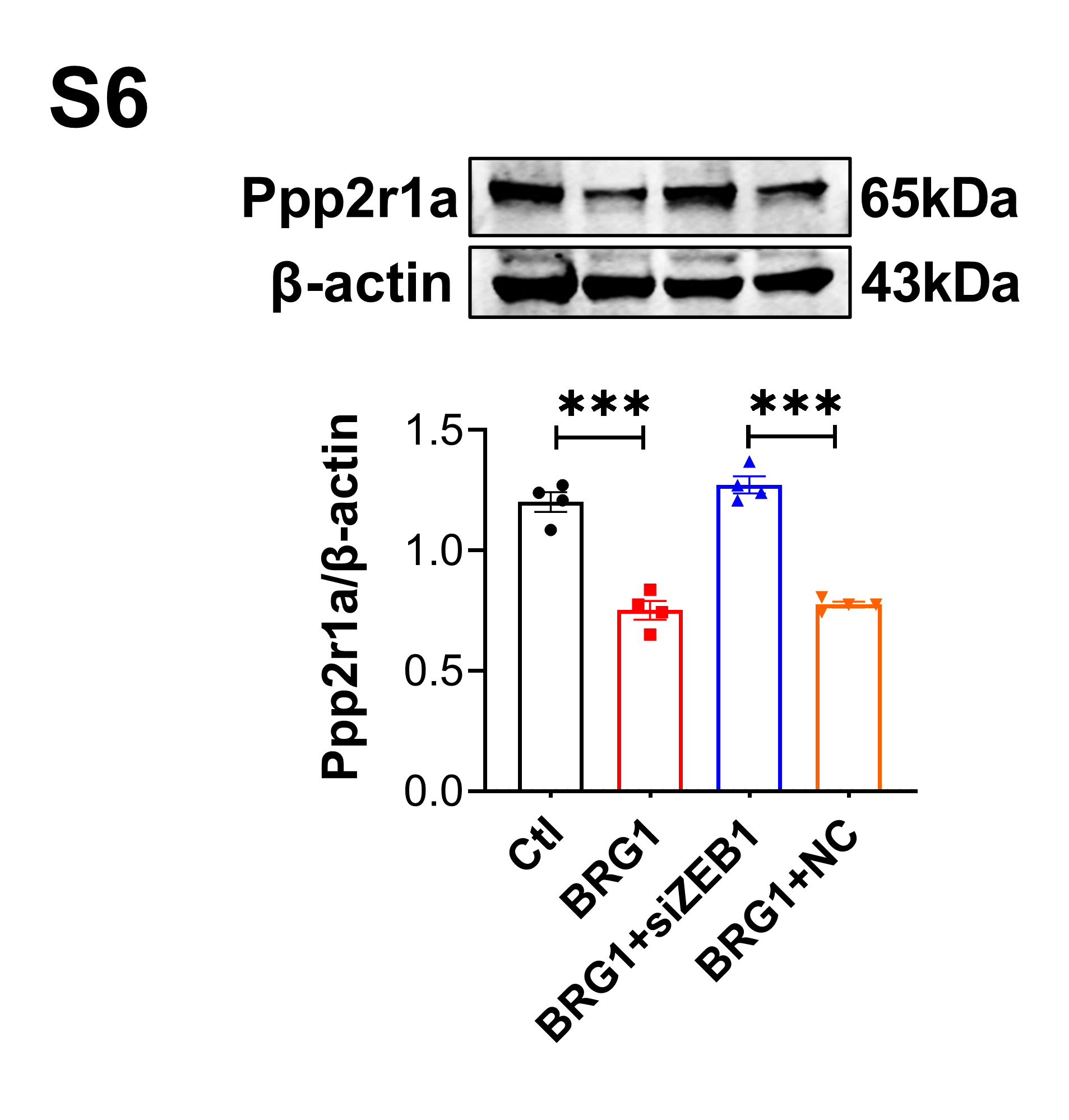


**Supplementary Figure 6. ZEB1 knockdown rescued BRG1-induced downregulation of *Ppp2r1a*, related to Figure 8.** Western blot analysis of Ppp2r1a protein levels in CFs following co-transfection with BRG1 overexpression plasmids and siZEB1. ****p* < 0.001 vs. Ctl group or BRG1+NC group by one-way ANOVA followed by Tukey’s post hoc analysis. n = 4.


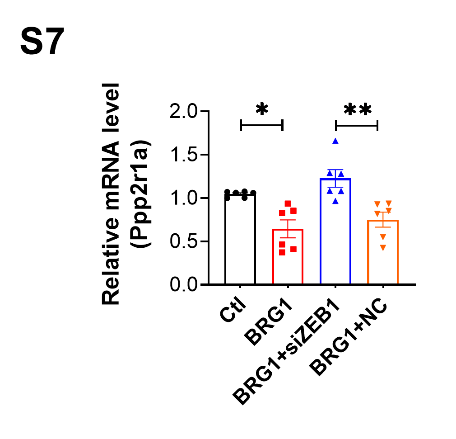


**Supplementary Figure 7. ZEB1 knockdown rescued BRG1-induced downregulation of *Ppp2r1a*, related to Figure 8.** *Ppp2r1a* levels in CFs co-transfection with BRG1 overexpression plasmids and siZEB1 by qRT-PCR. **p* < 0.05, ***p* < 0.01 vs. Ctl group or BRG1+NC group by one-way ANOVA followed by Tukey’s post hoc analysis. n = 6.


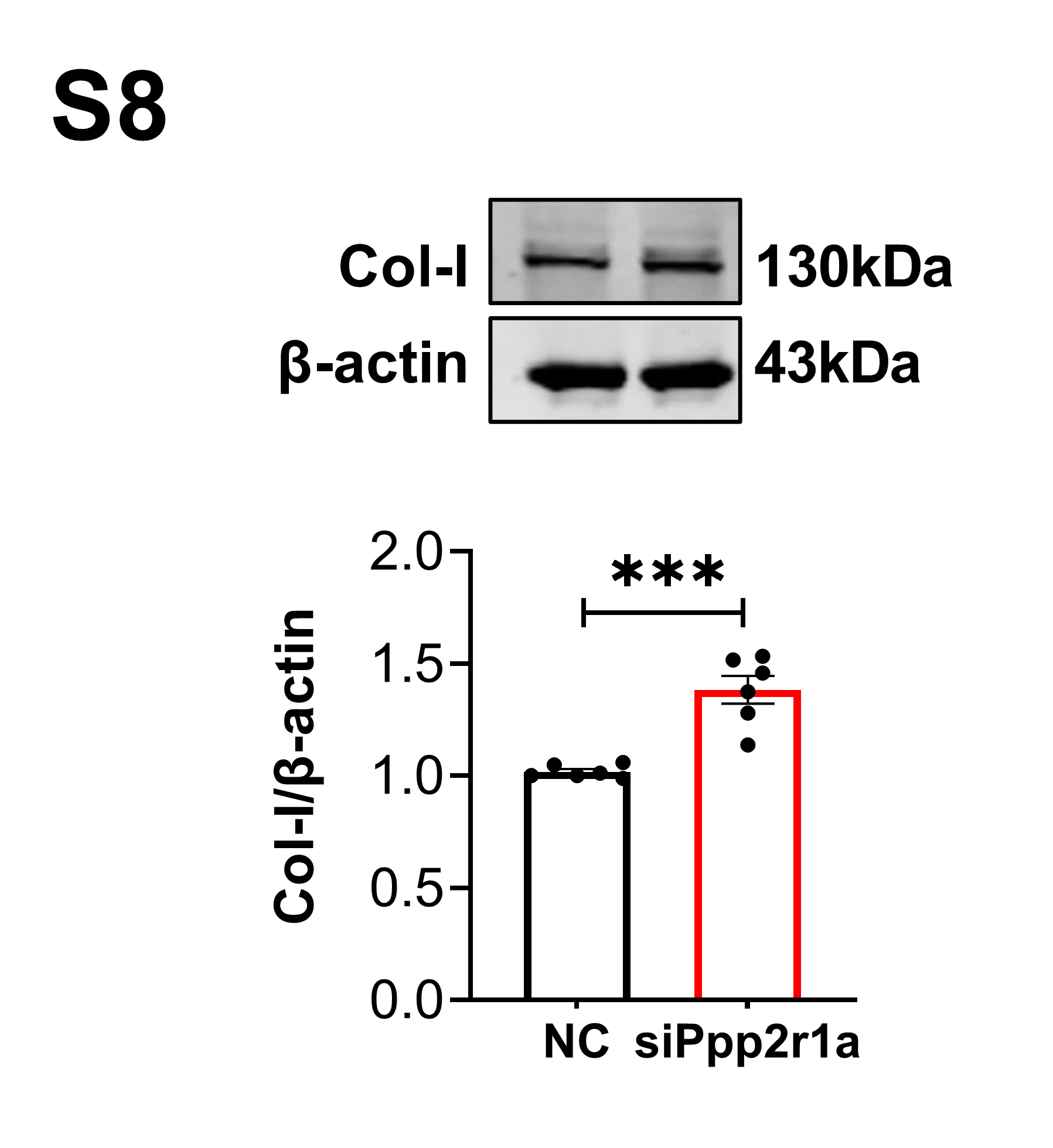


**Supplementary Figure 8. Knockdown of *Ppp2r1a* upregulated** **Col-I protein in CFs, related to Figure 8.**
Western blot analysis of Col-I protein expression following Ppp2r1a siRNA transfection in CFs. ****p*< 0.001 vs. NC group by a two-tailed Student’s *t*-test. n = 6.


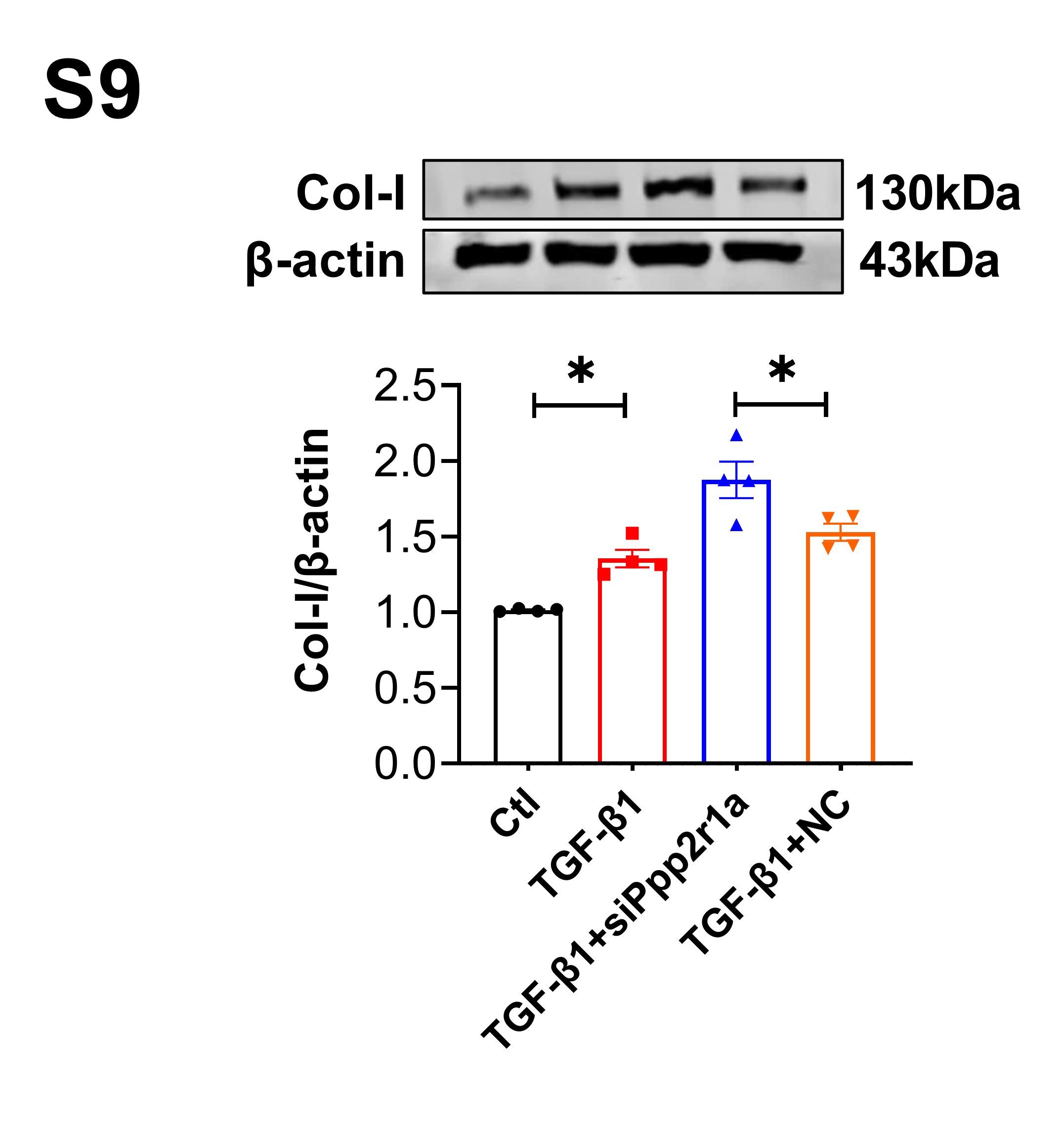


**Supplementary Figure 9. *Ppp2r1a* deficiency exacerbated the pro-fibrotic response to TGF-β1, related to Figure 8.** *Ppp2r1a* knockdown exacerbated TGF-β1-induced increase in Col-I protein level. **p* < 0.05 vs. Ctl group or TGF-β1+NC group by one-way ANOVA followed by Tukey’s post hoc analysis. n = 4.

**Supplementary Table 1. The sequences of primers for Quantitative real-time** RT-**PCR.**

| BRG1 | Forward | 5'-GGTTCTGCCCACAGCATGAT-3' |
| --- | --- | --- |
|  | Reverse | 5'-GGACTCCATAGGCTTGTGCAT-3' |
| ZEB1 | Forward | 5'-AATGCGGGAAGGCCTTCAAGT-3' |
|  | Reverse | 5'-ACTGAGATGTCTTGAGTCCTG-3' |
| Col-I | Forward | 5'-AAGAAGACATCCCTGAAGTCA-3' |
|  | Reverse | 5'-TTGTGGCAGATACAGATCAAG-3' |
| FN1 | Forward | 5'-CGAGGTGACAGAGACCACAA-3' |
|  | Reverse | 5'-GACACAACAATGCTCCCGA-3' |
| Ppp2r1a | Forward | 5'-TGCTGAGGACAAGTCTTGGCGT-3' |
|  | Reverse | 5'-TCATCAGGTTCTGGAAGGCAGG-3' |
| GAPDH | Forward | 5'-GGGGCTCTCTGCTCCTCCCTG-3' |
|  | Reverse | 5'-CGGCCAAATCCGTTCACACCG-3' |

**Supplementary Table 2. The sequences of siRNAs.**

| siPpp2r1a | sense | 5'-GACAGGAUAUCACCACCACCAA-3' |
| --- | --- | --- |
| siBRG1 | sense | 5'-GAGCGAATGCGGAGGCTTA-3' |
| siZEB1 | sense | 5'-GCAGTTACACCTTTGCATA-3' |
| hsiBRG1 | sense | 5'- GGUCAAUGGUGUCCUCAAA -3' |
